# Metabolic Signatures of Breast Cancer Subtypes and the Metabolic Impact of Chemotherapy

**DOI:** 10.3390/metabo16010054

**Published:** 2026-01-08

**Authors:** Aubrey Mattingly, Zoe Vickery, Alex Fiorentino, Ethan Wilson, Sydney McCune, Sydney Clark, Eric Blanchard, Jillian Spencer, Abigail Broom, Diana Ivankovic, Brooklyn Pace, Lauren Baskin, Ludovico Abenavoli, W. Jeffery Edenfield, Ki Chung, Christopher L. Farrell, Hakon Hakonarson, Luigi Boccuto

**Affiliations:** 1 Healthcare Genetics Laboratory, School of Nursing, Clemson University, Clemson, SC 29634, USA; 2 Center for Cancer Research, Anderson University, 316 Boulevard, Anderson, SC 29621, USA; 3 Department of Health Sciences, University “Magna Graecia”, Viale Europa-Germaneto, 88100 Catanzaro, Italy; 4 Prisma Health Cancer Institute, Greenville, SC 29605, USA; 5 Center for Applied Genomics, Children’s Hospital of Philadelphia, Philadelphia, PA 19104, USA

**Keywords:** breast cancer, metabolomics, metabolic profiling, chemotherapy, metastatic, primary, triple-negative, hormone receptor-positive, therapeutic tailoring, precision medicine

## Abstract

**Background/Objectives**: Breast cancer is a prevalent and heterogeneous disease with multiple subtypes, which are defined by characteristics such as molecular biomarkers and metastatic status. This study aimed to profile the metabolic activity of various breast cancer subtypes, both with and without chemotherapy (doxorubicin) application. **Methods**: Six human breast cell lines were evaluated, two non-tumorigenic controls and four cancerous lines. The cancer lines were clustered as primary-derived, metastasis-derived, triple-negative (TNBC), and strong hormone receptor-positive (ER+/PR+) and analyzed using the Biolog phenotype mammalian microarrays (PM-M1 to PM-M8) to assess metabolic activity via NADH production under a wide array of substrate parameters. **Results**: Unique metabolic profiles emerged across the subtypes and clusters; the TNBC and metastatic cells demonstrated enhanced utilization of glycolytic and anaerobic substrates consistent with the Warburg effect. The ER+/PR+ cells showed heightened glucose utilization and unique sensitivity to metabolic effectors and doxorubicin. Additionally, significant metabolic differences were observed in nucleoside and amino acid utilization between cancer and control cells, particularly in metastatic and TNBC lines. **Conclusions**: Our findings reveal the profound metabolic diversity among breast cancer subtypes and highlight distinct substrate dependencies for proliferation. The results additionally provide a framework for developing metabolic biomarkers and targeted therapies for chemotherapy resistance in breast cancer subtypes.

## 1. Introduction

Breast cancer is responsible for the most cancer diagnoses and deaths in women globally; its incidence is influenced by factors such as higher hormone levels, increased age, and the presence of genetic variants, including risk factor hotspots like *BRCA1* and *BRCA2* variants [1]. Breast cancer can be classified based on the expression of hormone receptors and other molecular markers. Major subclassifications include hormone receptor-positive (HR+) lines, HER2-positive (HER2+) lines, and triple-negative breast cancer (TNBC) lines. HR+ lines include cell lines that express estrogen receptors (ER+) and/or progesterone receptors (PR+). HER2+ cell lines overexpress the human epidermal growth factor receptor 2, which promotes tumor growth, and TNBC lines lack ER, PR, and HER2 expression. These biomarkers can influence the rate of metastasis, treatment resistance, and recurrence [2]. Breast cancers are also classified based on localization status into primary or metastatic, depending on whether the proliferation is confined to the initial site, with no sign of infiltration of neighboring tissues, vascular or lymphatic vessels, or if cancerous cells have spread to distant sites [3].

Current standard treatments for breast cancer include surgery, radiation therapy, endocrine therapy, immunotherapy, and chemotherapy [4]. These treatments may be used individually or in combination with one another, often in a sequential manner. For instance, the use of radiation therapy after surgery, followed by long-term hormone therapy [5]. The classification of breast cancer largely determines the most appropriate treatment strategy.

Surgical options for breast cancer include lumpectomy, which is a breast-conserving method that requires the tumor size to be below a certain threshold [6]. Other surgical options include unilateral or bilateral mastectomy, which involves the complete removal of breast tissue from one or both breasts, respectively. One study found that surgery involving primary tumor excision in patients with concomitant metastatic breast cancer reduces the mortality rate by 37% [7]. Many breast cancer patients will undergo a course of radiotherapy after breast surgery to improve locoregional control and overall survival. There are indeed risks associated with radiotherapy, including cardiac disease, radiation pneumonitis, lymphedema, and secondary malignancy [8]. Because estrogen and progesterone are key regulators of breast tissue growth and differentiation, they are targets in breast cancer treatment for hormone-dependent types [9]. Endocrine therapy or hormone therapy (HT) is a common and reportedly effective treatment for ER+ and PR+ breast cancers; however, it is also associated with certain varying risks, depending on the drug and mechanism of action. Options for estrogen blockers include pure antiestrogen drugs such as fulvestrant, as well as selective estrogen receptor modulators (SERMS) like tamoxifen [10]. Use of fulvestrant can cause respiratory issues, gastrointestinal distress, general bodily weakness and musculoskeletal pain, and loss of appetite, amongst other side effects. Tamoxifen carries a more severe set of dangers upon administration than fulvestrant, including the risk of developing hypercoagulability, cerebrovascular accidents, reduction in bone mass in premenopausal women, and second primary cancers in reproductive tissues, including uterine sarcoma and endometrial cancer [10].

In postmenopausal women, aromatase inhibitors prohibit the production of estrogen in the ovaries and can be quite effective for hormone-dependent cancers. In premenopausal women, aromatase inhibitors are not typically used unless the ovaries are suppressed with an additional drug as the quantity of estrogen produced in the ovaries is too high [10]. With the use of aromatase inhibitors and ovarian suppression, the adverse effects can include depression, a decrease in bone mass, and even cardiac issues, including heart attacks or heart failure [10]. Another consideration is the common use of hormone replacement therapy (HRT) for the alleviation of menopause symptoms, supplementing hormones instead of blocking them. It is not an endocrine therapy in direct relation to the primary breast cancer. This is important because menopause is inevitable in females and significantly increases the risk of breast cancer events in both survivors and healthy women. This increased risk is linked to the molecular characteristics of hormone-dependent cancers and the effects of adjuvant therapies used to treat or prevent them.

One study found that breast cancer survivors who took continuous hormone replacement therapy, specifically estrogen-progestin therapy to treat menopause, had an increased risk of recurrence [11].

A characteristic of cancer is its capability to evade the immune system, which has led to the development of tumor-targeting immunotherapies, oncolytic viruses, and anticancer vaccines [12]. Immunotherapy can also lead to immune-related adverse events (IRAEs) such as endocrinopathies, arthritis, xerostomia, and neurotoxicity [13]. Chemotherapy is a common breast cancer treatment method involving the use of cytotoxic chemicals to target cancer cells. Chemotherapeutic agents like doxorubicin (Adriamycin) result in potential adverse effects, including nausea, fatigue, and immune suppression [14], with the potential for more severe and life-threatening side effects, such as cardiovascular complications. Although these treatment methods represent the current standard of care, alternative methods are being investigated due to their potential to reduce harmful side effects. One alternative treatment option under investigation is the use of nutraceuticals or herbal remedies for their potential to selectively target cancer cells with less pronounced effects on healthy cells [15].

Due to the heterogeneity and complexity of cancer, it is critical to decipher molecular processes, including the cellular and physiological activities that promote energy production and cancer proliferation. This allows for treatment identification strategies to target and inhibit along these processes, which are unique to cancer cells, particularly with different molecular features, tissue types and locations. Metabolomic approaches have been particularly efficacious at identifying cancer-specific alterations by investigating metabolites and small molecules in specimens directly or indirectly connected to the cancerous cells. Metabolomic findings can be instrumental in indicating prognosis, therapeutic targets, and diagnostic markers by measuring phenotypic changes that reflect genetic alterations [16]. In breast cancer specifically, metabolomics has been used to distinguish between subtypes. One study demonstrated that ER+ and HER2 breast cancers differ in their glutamate–glutamine ratios as well as levels of aerobic glycolysis [17]. Another study identified three metabolic subgroups within TNBC based on dependency on lipid metabolism and glycolysis [18]. Additionally, metabolomics has been employed to evaluate the effects of breast cancer treatment. For instance, treating MCF-7 breast cancer cells with doxorubicin, as compared with treatment with a plant extract, marjoram, revealed marjoram’s phytochemical potential to regulate key metabolic effectors [19]. The field of metabolomics is rapidly expanding and holds significant potential for cancer research. In this study, it has been utilized to gain deeper insight into the metabolic state of different breast cancer cell lines.

We hypothesized that breast cancer subtypes exhibit distinct metabolic profiles that can be modulated by chemotherapy. This study investigated the metabolic signatures of multiple breast cancer subtypes, including primary and metastatic lines and healthy fibrocystic breast tissue controls modeled in vitro. Our experimental objective was to elucidate metabolic profiles of human breast cancer overall, as well as in various clusters, without treatment and with a traditional chemotherapeutic reagent, doxorubicin. Clustering includes localization features using clinical definitions between primary and metastatic sites of cellular origin, as well as molecular features, using receptor status similarities to analyze molecular marker parameters. Potential therapeutic targets may emerge from the elucidation of the effects of energy production, enhancement, or disruption, as well as cellular resource prioritization, in the presence of specific chemicals. Furthermore, we aimed to investigate how the metabolic environment influences the cellular response and cytotoxicity of breast cancer cells through energy production profiles under normal conditions and following exposure to a chemotherapeutic agent.

## 2. Materials and Methods

### 2.1. Cell Lines Selected and Media

Six adherent glandular epithelial breast cell lines were utilized for metabolic profiling and cellular activity monitoring in this study. Two of the six lines, MCF-12A and MCF-12F which are adherent non-tumorigenic epithelial breast cells, were used as controls. These cell lines exhibit regular luminal epithelial morphology and are derived from the breast tissue of patients with fibrocystic breast disease. Two primary tumor-derived cell lines were used for the breast cancer cell lines. HCC-38 was derived from a primary ductal carcinoma in the mammary gland, but, notably, it is very invasive for a primary line. This is estrogen receptor negative (ER−), progesterone receptor negative (PR−), and human epidermal growth factor receptor 2 negative (HER2−), also known as a triple-negative breast cancer (TNBC) cell line. BT-483 was derived from a papillary invasive ductal carcinoma and is less aggressive than HCC-38, with its hormonal features being that it is ER+/PR+ but negative for HER2. The last two cell lines used are established from metastatic breast cancers. HTB-26 cells originate from a metastatic adenocarcinoma and present a triple-negative profile (ER−/PR−/HER2−). This cell line in the study is referred to by its accession number (HTB-26) instead of MDA-MB-231. MCF-7 cells are among the most widely used cell lines in breast cancer research and exhibit aggressive metastatic behavior. The MCF-7 cell line was derived from a pleural effusion metastatic site with retention of some characteristics of differentiated mammary epithelium and is ER+/PR+ but HER2−. Table 1 summarizes the cell lines and their relevant features, as used for clustering in this study.

Clustering was assigned (Table 2) based on the American Type Culture Collection (ATCC) clinical definitions of anatomical origin, including primary breast tumor-derived lines, and which are separate from those of metastatic origin. The molecular features were also used to create cluster subgroups, including luminal cell lines that are strongly hormone receptor positive and TNBC basal-like/low-differentiated luminal models.

Each cell line was maintained in complete growth medium, which provided proper nutrition and consisted of the specific base medium for the cell type, supplemented with additional components. Growth medium is essential for successful cell culture, as its contents provide proper nourishment as well as protection from bacterial contamination. The base medium is specified on the manufacturer’s handling information and can be found via the ATCC. HTB-26 requires Leibovitz’s L-15 medium. Both BT-483 and HCC-38 utilize RPMI-1640 medium. MCF-7 uses Eagle’s minimum essential medium (EMEM). For MCF-12A, Dulbecco’s minimum essential medium (DMEM) with F-12 is used. For MCF-12F, both DMEM and Ham’s F-12 base are required. For this study, the complete growth medium for each cell line was created with the following ingredients: 500 mL of the specific base medium for each cell line, 50 mL of fetal bovine serum (FBS), and 5.0 mL of penicillin–streptomycin. All ingredients were added aseptically to the 0.1 μm filtration system and vacuum filtered. Once all ingredients were properly filtered, the bottle was labeled as complete and stored in the refrigerator.

### 2.2. Thawing Cells from –80 °C

Cell lines were purchased from ATCC, received in cryovials packed in dry ice, and stored immediately in cryo-storage if not used upon receipt. Per the manufacturer’s handling procedures, the cells were thawed upon or shortly after their receipt with minimal time in the freezer. Thawing was performed by partial submersion in a water bath at 37 °C, ensuring that the O-ring and cap remained above the water level. The vials were removed from the water bath once the crystals had disappeared, decontaminated with a 70% ethanol spray, and placed under the biosafety cabinet. The procedure then continued in a sterile environment. Cryopreservation media was removed by spinning down the cell pellet and aspirating the supernatant. The cells were resuspended in 5.0 mL of complete growth media, warmed to room temperature, and placed in a T-25 cm^2^ adherent, sterile flask to allow for cell acclimation and growth. The cells were then incubated for 48 h within the flask(s) before the old media was replaced to ensure that cell membranes and adhesion were not disrupted from centrifugation or other cell passaging techniques.

### 2.3. Cell Culturing and Passaging

All cells were cultured in T-75 cm^2^ adherent, sterile, cell culture flasks in an incubator at 37 °C with 5% flowing CO_2_, except for HTB-26 cells, which require 0% flowing CO_2_ and instead 100% O_2_ at the same temperature. The cells were fed consistently, meaning the complete growth medium was replaced every 48–72 h to ensure the cells had proper nutrients for growth. For feeding, the old complete growth medium was removed from the flask and discarded into a waste beaker. Then, 15.0 mL of fresh complete growth medium (warmed to approximately 37 °C) was added to the cell culture flask. All old complete growth medium was neutralized with bleach prior to disposal. Cell cultures were passaged once they reached approximately 70–75% confluency. Passaging began with removing the old top medium from the cell culture flask, adding 5 mL of PBS to rinse the cells, and aspirating it into waste. Next, 5.0 mL of 0.25% Trypsin EDTA (warmed to approximately 37 °C) was added to incubate on top of the cells to detach the cell membranes from the flask. After the addition of trypsin, the flasks were gently shaken to ensure complete coverage and incubated at 37 °C for approximately 5 min. After incubation, complete medium was added to the flask at a volume of 5.0 mL or more, ensuring a 1:1 ratio of trypsin and media to quench/inhibit the activity of the proteases. The cell suspension containing trypsin and the neutralizing media was removed from the flask and placed into a 50 mL conical tube for centrifugation at 1000 RPM for 5 min. The supernatant was discarded, and the cell pellet was resuspended in 15.0 mL of new complete growth medium (warmed to approximately 37 °C). Cultures were maintained at sub-cultivation ratios of 1:2 or 1:3, depending on growth rate. All liquid waste was neutralized with bleach prior to disposal in the sink.

### 2.4. Trypan Blue Exclusion Assay

The Trypan Blue exclusion assay provides vital information on cell viability, including the percentage of live/dead cells, as well as the concentration of live cells per 1.0 mL of solution. The information and performance of this assay were typically obtained after resuspension prior to sub-cultivation, during passaging of each line, and before all plating in the microarrays to ensure proper cell counts, as described in the subsequent methodology Section 2.5. For this assay, 20 μL of resuspended cell solution was added to a 2.0 mL Eppendorf tube containing 20 μL of 0.4% Trypan Blue dye (1:1 ratio of cells to dye). Dead cells absorb the dye, becoming dark, while live cells remain lighter in color. Once the cell solution and dye were properly mixed within the Eppendorf tube by pipetting up and down, 10 μL of the solution was added to each side of a cell counting chamber slide. Each side of the slide was then inserted, one at a time, into a Countess III Automated cell counter to obtain the percentage and concentration of viable cells within the solution. The data from each side were then averaged.

### 2.5. Biolog Phenotype Mammalian Microarrays

Biolog phenotype mammalian microarrays (PM-Ms) [22] are 96-well microplates that allow for the fingerprinting of mammalian cells’ metabolic signature. They are designed for testing metabolic profiles, and the information they generate is interpreted regarding pathway utilization through measurements of cells’ energy production in various environments. Cellular energy production, using the reduced form of nicotinamide adenine dinucleotide (NADH), is assessed by a colorimetric reagent (tetrazolium dye) that is reduced to a purple formazan, darkening as redox energy is produced from the oxidation of a chemical. Each well contains an energy source (PM-M1 through PM-M4) or metabolic effector (PM-M5 through PM-M8). There are 367 potential metabolic pathways tested in PM-M1 through PM-M4. In PM-M5 through PM-M8, the metabolic effectors coating the wells are designed to elucidate the effects of the chemicals on the growth, productivity, and metabolism of mammalian cell lines. Examples of chemicals coated in the microplate wells include carbon energy sources (PM-M1), amino acids acting alone and as dipeptides (PM-M2 through PM-M4), ions (PM-M5), hormones, stimulants, growth factors, and cytokines (PM-M6 through PM-M8).

The media included 1.1 mL of 100X penicillin/streptomycin solution to prevent bacterial growth and contamination, 0.16 mL of 200 mM glutamine to achieve a final concentration of 0.3 mM, and 5.3 mL of fetal bovine serum (FBS) to achieve a final concentration of 5%. This is added to 100 mL of inoculating fluid (IF-M1). The Biolog IF-M1-modified medium was used for all of the wells. Plates 1 through 4 are seeded with complete media containing FBS, and plates 5 through 8 contain the addition of glucose substituted for FBS. The glucose serves as the sole source of energy for the cells seeded in these plates, at a final concentration of 5%, using 5.5 mL of a 100 mM stock solution. For one PM-M plate, 2,000,000 cells are used in a volume of 5 mL of liquid (40,000 cells/mL). This is dispensed into each well, resulting in a final volume of 50 μL of media plus cells, with 20,000 cells per well. Cells were used between passages 10 and 15 to ensure viability and minimize phenotypic drift. For the experimental set with added traditional chemotherapeutics, 10 μL of 0.5 μM doxorubicin was mixed into the cell suspension. The concentration of doxorubicin was determined based on the manufacturer’s recommendations (https://www.cellsignal.com/products/activators-inhibitors/doxorubicin/5927. Accessed on 2 June 2022). We selected the lowest end of the suggested concentration range, as our aim was to explore the metabolic effects of the drug without killing the cells. Once the cells, media, and doxorubicin (if applicable) were plated into PM-M plates 1–8, the cells were incubated for 48 h, during which the only available energy source to the cells is the chemical coated in the well. The environmental conditions for this first 48 h incubation were 37 °C in 5% CO_2_ (apart from HTB-26, which was incubated in 100% air).

Following the 48 h incubation, Biolog Redox Dye Mix MB was added quickly under the fume hood in a dark room (with a multichannel pipette) at a volume of 10 μL per well. The 96-well microarray plates (with the redox dye added) were immediately placed into the Omnilog^®^ machine (Biolog Inc, Hayward, California, USA) under the same incubation conditions for 24 h. During this time, NADH produced in the wells reduced the purple formazan by metabolizing the sole energy source. Darker wells indicated higher NADH production for cells during their incubations. The endpoint readings at 590 and 750 nm were captured at precisely 24 h following the dye incubation using a microplate reader. The first value, A_590_, provided the highest absorbance peak of the redox dye. The second value, A_750_, represented a measure of the background noise that was subtracted from the highest absorbance, outputting the relative absorbance per well (A_590_–A_750_).

### 2.6. PhenoMetaboDiff and Statistical Analysis

The raw data generated by the Biolog system that were used for analysis include an endpoint value of relative absorbance for each well in the PM-M plate, collected by the plate reader after incubation. To minimize or eliminate biases due to colorimetric responses independent from the cellular metabolism, all of the raw values were normalized versus the average of three experiments conducted with only media and dye in the plates (no cells). The data generated from this empty-cell experiment were considered as the non-specific colorimetric value against which all our data were normalized. Each cell line condition and plate was run in triplicate to standardize results; average normalized values were calculated for triplicate experiments. The primary metric for comparison is the mean normalized absorbance per well, from triplicate runs, for each group in the experiment.

For data processing and computational analysis of the collected microarray data, R Studio (R v4.1.2) was used with the PhenoMetaboDiff (v1.0.0) open-source software package. For implementation, the code is available online at https://anonymous.4open.science/r/phenoMetaboDiff-62D4 (Accessed 1 June 2025). With the deployment of PhenoMetaboDiff (PMD), this package enables the high-throughput identification, visualization, and statistical analysis of metabolomic data from the Biolog PM-M system. The sample comparisons conducted in PMD utilize the Mann–Whitney U test and obtain Benjamini–Hochberg (BH) adjusted *p*-values. The Mann–Whitney non-parametric U test is a more conservative option than the Student’s *t*-test for small sample sizes and does not require normal distribution of the samples [23]. The Benjamini–Hochberg method is a common approach to adjustment and is statistically sound for the multiple comparisons setup in this experimental framework, where many hypotheses are simultaneously tested. It adjusts the *p*-values, controlling for type I errors and false discovery rates (FDRs), increasing statistical power [24].

The normalized endpoint data, after averaging, were transposed to columns and set up for “patient” corresponding with the cancer cell lines (HCC-38, BT-483, HTB-26, and MCF-7) and “control” corresponding with the control lines (MCF-12A and MCF-12F). The file format was exported to CSV to accommodate the import utility of the PhenoDiff module. After importing, the plate number was selected, and the patient and control columns were assigned. The analysis output revealed significant differences in the energy utilization of metabolites (NADH production) between the two groups, specifically related to the plate number. The identification of “true” significance was set at a threshold of α = 0.05, with a *p*-value < 0.05 resulting in a highlighted row for that well. Significance was defined as a BH-adjusted *p*-value < 0.05. The highlighted row indicated that the patient and control means were significantly different following BH adjustment.

**Figure 1:**
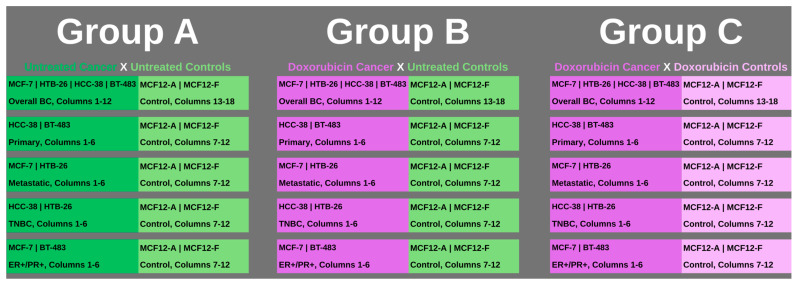
The groups for multiple comparisons and analysis are outlined. Group A covered all of the established clusters with both cancer and control cell lines with no treatment applied (represented in darker green for cancer cell columns and lighter green for control cell columns). Group B also covered all of the established clusters, with the cancer cell lines having chemotherapy, specifically doxorubicin, applied (represented in purple), while the comparison control columns had no treatment (represented in lighter green). Group C covered all of the established clusters with both cancer and control cell lines having chemotherapy, doxorubicin, applied (represented in purple for cancer cell columns and control cell columns). All clusters within groups had triplicate repeats per cell line and were conducted for all plates, 1 through 8.

To visualize the multiple comparisons set up for analysis within the overall groups and clusters, as well as those involving doxorubicin, Figure 1 below depicts the layout for reference.

## 3. Results

We employed the Biolog PM-M technology to study the energy production of cancerous and control breast cells in different metabolic environments. The PM-M assays allowed us to investigate how the tested cell lines utilized different carbon-based energy sources, such as carbohydrates, carboxylic acids, intermediates of the Krebs cycle (PM-M1), amino acids and dipeptides (PM-M2, M3, and M4) to generate NADH. The assays also enabled us to assess how the same cells utilize glucose to produce NADH in the presence of ions (PM-M5), growth factors, hormones, cytokines, and other metabolic effectors (PM-M6, M7, and M8).

The complete set of results can be accessed in the Supplementary Materials. Group A corresponds to five sheets for clustering, each with eight pages per sheet for the PM-M plate in Supplementary Materials. The same setup applies to Group B in Supplementary Materials and Group C in Supplementary Materials. All of the output analysis sheets were used for data processing, depicting significance, tabulating wells, and presenting original normalized absorbance values from triplicate runs, organized according to the clustering for multiple comparisons.

### 3.1. Untreated PM-M1 Plate (Carbon Energy Sources)

Chemicals coated on the first PM-M plate are those relevant to glycolytic substrates, including both glucose derivatives and phosphorylated forms, which are important for understanding the shift in metabolism between aerobic and anaerobic conditions in cancer cells. Analysis of carbon source utilization (PM-M1) revealed distinct patterns consistent with the Warburg effect, particularly in TNBC and metastatic clusters. The substrates that showed particularly informative differences included D-(+)-glucose (wells B4, B5, and B6), D-glucose-6-phosphate (B1), D-glucose-1-phosphate (B2), D-fructose-6-phosphate (D6), D-fructose (D7), glycogen (A6), dextrin (A5), D,L-lactic acid (G2), pyruvic acid (G5), L-malic acid (G10) and D-malic acid (G11).

D-(+)-glucose is the input for glycolysis, the consistent classical energy source in the wells. The only cluster with statistically significant differences in energy production from the D-(+)-glucose wells was the ER+/PR+ lines versus controls. Two wells (B5, B6) showed significantly higher energy production in these cancer cells both before and after *p*-value adjustment, while the difference in the third well (B4) followed the same trend but was statistically significant (adjusted *p* = 0.006).

The overall BC signature cluster produced significantly higher NADH levels than controls in the presence of glycogen, the stored form of cellular glucose, while the ER+/PR+ and primary clusters showed a similar trend but reached significance only for the unadjusted *p*-values (both at 0.004). Dextrin, an intermediate of glycogen digestion, showed a statistically significant increase in energy production across multiple clusters and diverse cancer subtypes, with adjusted *p*-values of 0.03, 0.03, and 0.02 for TNBC, metastatic and overall BC, respectively. Although the primary lines showed no significance when adjusted, it is worth mentioning that they were relatively close to the threshold, with an unadjusted *p*-value of 0.004.

Both TNBC and metastatic clusters produced more NADH than controls utilizing D,L-lactic acid as an energy source (adjusted *p* = 0.02 and 0.02, respectively). This finding indicates an increased anaerobic respiration activity as lactic acid is a final product of the anaerobic route of glycolysis. Pyruvic acid (G5) is a pivotal metabolic biomarker that serves as a precursor in both the aerobic (acetyl-CoA) and anaerobic (lactate) pathways of cellular respiration. The metastatic cluster was the only one to detect a significantly higher utilization of this substrate (adjusted *p* = 0.01). Both the TNBC and the metastatic clusters produced higher NADH levels than controls in the presence of L-malic acid (G10), a Krebs cycle intermediate, and D-malic acid (G11): the adjusted *p*-value was 0.03 for both wells in TNBC and 0.02 for L-malic acid and 0.01039 for D-malic acid in the metastatic group.

An interesting trend was the increased energy production in cancer cells in the presence of nucleosides: Thymidine (E9), uridine (E10), adenosine (E11), and inosine (E12). These molecules are well recognized as precursors for critical processes, including DNA and RNA synthesis. In the metastatic cluster, thymidine, uridine and adenosine showed significant differences as compared with controls (adjusted *p* = 0.02, 0.01, and 0.01, respectively). Significantly higher energy levels were generated by thymidine and adenosine in the overall BC signature (both adjusted *p* = 0.02), thymidine alone in the TNBC group (adjusted *p* = 0.03), and adenosine in the ER+/PR+ group (*p* = 0.04). No significant nucleoside-coated well emerged in the primary cluster.

### 3.2. Doxorubicin-Treated PM-M1 Plate (Carbon Energy Sources)

We tested the cancer cells’ ability to produce energy on PM-M1 after exposure to doxorubicin as compared with untreated controls (Group B) and doxorubicin-treated controls (Group D). Analysis of carbon source utilization (PM-M1) with doxorubicin treatment revealed patterns consistent with cytotoxicity and severe inability for all cell types to produce energy. In Group B, the cancer cells across all clusters appeared to be incapable of producing energy in the presence of the PM-M1 substrates, with all plates showing decreased energy production compared with untreated controls, demonstrating significance in 88% or more of the wells. In the TNBC group, all PM-M1 wells showed significantly reduced NADH levels. In Group C, there was no significant difference in energy production between doxorubicin-treated control cells and the cancer cells in the overall BC, metastatic, and TNBC clusters. The primary and ER+/PR+ cells generated significantly lower energy levels in controls in approximately one-third of the PM-M1 wells, including classical energy sources and early steps of glycolysis, like all three wells of D-(+)-glucose, D-glucose-6-phosphate, D-glucose-1-phosphate, glycogen, dextrin, and in the primary cluster only, D-fructose-6-phosphate, and D-fructose.

### 3.3. Untreated PM-M2, PM-M3, and PM-M4 Plates (Amino Acid Derivatives)

Analysis of amino acid derivative utilization (PM-M2–4) revealed patterns consistent with metabolic rewiring and less efficient energy utilization/protein synthesis, particularly in TNBC and some in metastatic clusters. PM-M plates 2 through 4 are coated with amino acids on their own as well as in dipeptide forms. Metastatic cluster cells produced higher energy levels than controls in PM-M2. The most represented amino acid in the significant wells for metastatic breast cancer cells was the dicarboxylic amino acid, glutamic acid. In the TNBC cluster, there was an extremely high number of significant wells with cancerous cells producing higher NADH in more than 50% of all wells of PM-M2, PM-M3, and PM-M4. The most frequently significant amino acids (present in 20 or more wells in dipeptide conjugations or individually) in TNBC were tryptophan, valine, tyrosine, leucine, and proline, in decreasing order of the number of significant wells.

### 3.4. Doxorubicin-Treated PM-M2, PM-M3, and PM-M4 Plates (Amino Acid Derivatives)

Untreated control cells produced consistently more energy than doxorubicin-treated cancer cells from any cluster in the Group B analysis, with 85% or more wells from plates PM-M2 to M4 reaching statistical significance. These data show, again, a constant inhibition by doxorubicin of the cancer cells’ ability to metabolize the energy sources to successfully grow and proliferate. In Group C, a pattern similar to the one seen for the clusters in PM-M1 was evident for the primary and ER+/PR+ groups. The analysis showed controls being significantly higher than the cancer cells in their ability to utilize approximately one-third of the substrates, in this case, amino acid derivatives, to generate energy. The overall BC, metastatic, and TNBC groups treated with doxorubicin did not show significant differences in these plates compared with the controls treated with doxorubicin. One interesting exception was on PM-M4 in the overall BC profile, where seven wells containing phenylalanine generated significantly higher NADH levels in controls.

### 3.5. Untreated PM-M5, PM-M6, PM-M7, and PM-M8 Plates (Metabolic Effectors)

The last group of plates contains molecules that induce metabolic responses, including ions, stimulants, steroid hormones, cytokines, and growth factors. The measurement of NADH produced is not from the utilization of the chemical as a source of energy, but instead shows how capable the cells are to metabolize glucose while the effector is also present. Each of the chemical substrates is represented in a range of six concentrations, outputting a unique data point per increasing concentration. The data revealed a distinct subtype in ER+/PR+ cancerous cells from PM-M5, characterized by metabolic disruption and reduced baseline activity. For primary cells on PM-M6 through PM-M8, the high volume of wells and significant substrates depict another metabolic disruption that is quite unlike any of the other cell lines. From the TNBC on PM-M6 and PM-M7, and metastatic clusters on PM-M5, a loss of feedback regulation is shown by a failure of cancer cells to respond to effectors and alter their metabolic rate, as they did for control cells, which are significantly higher in many wells.

### 3.6. Doxorubicin-Treated PM-M5, PM-M6, PM-M7, and PM-M8 Plates (Metabolic Effectors)

Group B resulted in significant wells across every group. To elaborate, the findings of significance showed that the untreated controls, cells without doxorubicin, produced significantly more NADH, approximately 90% or more, and even up to 100% of the energy produced. In Group C, the TNBC, metastatic, and overall BC clusters revealed no significant differences with doxorubicin-treated controls. Some differences were observed in PM-M7 only for the ER+/PR+ group in the wells containing modulators of glucose and lipid metabolism: all concentrations of insulin (wells A7–A12), resistin (B1–B6), glucagon (B7–B12), ghrelin (C1–C6), leptin (C7–C12), and one well of gastrin (D1). In a couple of the wells for this group on PM-M7, on either the lowest or highest range of concentrations (insulin (A12) and resistin (B01)), the cancer cells were able to produce more energy, whereas the general trend observed was that control cells were able to produce significantly more NADH than cancer cells. For the last cluster in Group C, the primary cell lines showed varying but significant effects on all of the metabolic effectors in PM-M plates. On PM-M3-8, at least one of the significant wells depicted more energy produced in the cancer cells’ direction than in the control cells. These wells included His-Glu (B7) on PM-M3, Phe-Ile (A5) on PM-M4, NaCl, 3-isobutyl-1-methylxanthine (B1) on PM-M6, and (Arg8)-vasopressin (A12) on PM-M8. Similarly, on PM-M7, the same cell metabolism modulators were significant as in the ER+/PR+ group (insulin and resistin). In PM-M5, certain ions, including NaCl (wells A5–A12), ammonium chloride (B1–B4), sodium selenite (B5–B8), potassium chloride (B9–B12), calcium chloride (C1–C4), and manganese chloride (C5 and C6), were significant in both directions. The majority of the significant wells showed higher energy produced in the control cells than in the patient cells, with some exceptions. A5 (NaCl), A6 (NaCl), A12 (NaCl), B1 (potassium chloride), C3 (calcium chloride) and C4 (calcium chloride) showed significantly higher energy production in the primary cancer cells. In PM-M6, three substrates that amplify cellular signaling and increase cAMP levels resulted in higher energy production by the control cells as compared with the cancer cells: dibutyryl-cAMP (A7–A12), 3-isobutyl-1-methylxanthine (B2–B6) and some of the caffeine wells (B7–B10). In PM-M8, two hormones with their full concentration ranges induced a significantly higher energy production in control cells: parathyroid hormone (B1–B6) and Arg8-vasopressin (A7–A11), with the exception of A12, which significantly increased energy production in cancer cells.

### 3.7. Diversity of Profiles

**Figure 2:**
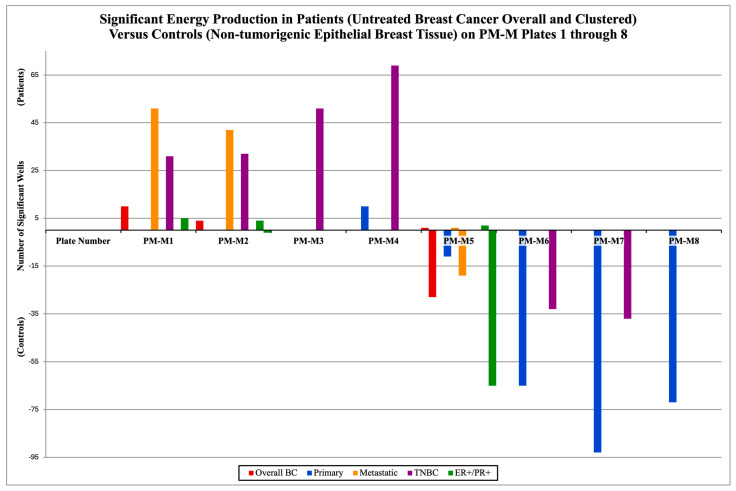
**Different metabolic signatures of breast cancer and within the clustered subtypes from group A, all without treatment.** The number of significant wells on each of the PM-M plates is shown above. If there were no significant wells, there would be no bar for that plate corresponding to a cluster. The positive values represent the mean cancer cells’ energy production (patients) being significantly higher than the controls’ (mean healthy cells’ energy production). The negative values represent the opposite; the controls’ NADH levels are significantly higher. All wells tallied in this figure were deemed significant compared with the previously established threshold (*p*-value < 0.05) after BH adjustment using the PMD software (v1.0.0).

The findings from the PM-M microarrays indicate that each cluster is characterized by a specific metabolic signature; Figure 2 depicts the energy production in cancer cells by clustering for plates PM-M1-8 for untreated cancer and untreated controls (Group A). The significance is evident in two directions, above and below the *x*-axis of the figure, representing both the NADH production of cancer cells and that of control cells. If the bar is above the *x*-axis, it represents cancer cells generating significantly higher levels of NADH in the corresponding plate. If the bar is below the *x*-axis, it represents the same output but from control cells.

In Group A, overall BC had cancer cells producing higher NADH levels in 10 wells on PM-M1, 4 on PM-M2 and 1 in PM-M5 (manganese chloride), while control cells generated higher levels in 28 wells in PM-M5 (28/96 = 29.2%). The primary cluster had cancer cells higher in 10 wells on PM-M4, and control cells were higher in 11 wells on PM-M5, 65 on PM-M6 (67.7%), 93 on PM-M7 (96.9%), and was the only cluster to show significance on PM-M8 with 72 wells (75.0%). The metastatic cluster had higher energy production by cancer cells in 51 wells on PM-M1 (53.1%), 42 on PM-M2 (43.8%), and 1 on PM-M5 (manganese chloride), while NADH levels were higher in control cells in 19 wells on PM-M5 (19.8%). TNBC had cancer cells higher in 31 wells on PM-M1 (32.2%), 30 on PM-M2 (32.3%), and was the only cluster to have significance on PM-M3, with 51 wells. On PM-M4, there were 69 significant wells for TNBC, with patient cells being higher (71.9%). TNBC had significantly higher energy production in control cells in 33 wells from PM-M6 (34.4%) and 37 wells from PM-M7 (38.5%). The ER+/PR+ cluster had cancer cells that were higher in five wells on PM-M1, four wells on PM-M2, while control cells were higher in one well (Arg-Leu). Additionally, control cells were higher in 65 wells of PM-M5, and cancer cells were higher in 2 wells (both containing manganese chloride).

## 4. Discussion

The PM-M assay results displayed a unique fingerprint for the metabolic activities of breast cancer cell lines. It enabled the overall profiling of breast cancer cells across multiple subtypes, compared with fibrocystic breast controls, a matched tissue type. Group A comparisons were conducted for all of the cancer cell lines without treatment, compared with all of the controls without treatment. This was undertaken for all lines combined and then for each cluster. The cancer cell lines were divided by clinically defined features from their origin location, including primary and metastatic cell lines, and further categorized by their molecular features, specifically examining triple-negative and double-positive receptor outlines. The analysis for Group B was conducted to stratify the metabolic profile of cancer groups based on their response to exposure to doxorubicin, as compared with untreated controls. Group C matched the cancer cells treated with doxorubicin with the control cells that underwent the same treatment. This allowed us to look at the clinical and molecular clusters as well.

The data from PM-M1 help depict the efficacy of aerobic and anaerobic metabolism through the utilization of glycolytic substrates, mitochondrial oxidative phosphorylation, and Krebs cycle-related intermediates. The analysis detected a significantly higher utilization of glucose-related substrates by cancer cells compared with controls. This trend is consistent with the Warburg effect, an extensively studied metabolic reprogramming observed in multiple cancer types [25], which involves a preferential use of glycolysis over aerobic pathways in the presence of oxygen. This shift was observed in all clusters of the present study and confirmed the tendency of cancer cells to prioritize less efficient metabolic pathways, thereby favoring faster energy production to sustain the elevated proliferative pace. Downstream glycolytic metabolites, including pyruvic acid, lactic acid and malic acid, also showed higher utilization in the cancer cells, specifically in metastatic and TNBC cells, a pattern consistent with the Warburg effect. In Group B, where control cells without treatment demonstrated significantly higher energy production than cancer cells with doxorubicin in the primary and ER+/PR+ groups, no significant differences were observed in these downstream glycolytic metabolites. This reveals that exposure to doxorubicin decreases the Warburg effect in cancer cells. The mechanism observed in the primary and ER+/PR+ groups involves the inhibition of the preferential utilization of glycolysis in cancer cells, and the restoration of traditional aerobic energy metabolism, which generates metabolic profiles characteristic of healthy cell growth and proliferation. The shift or metabolic rewiring described by the Warburg effect permits the “quantity over quality” nature of cancer growth, as cells are quickly multiplying while only building a minimal (still functional but not efficient) infrastructure for invasion and growth; the metabolic alterations in cancer cells are also allowing them to potentially move through areas where there is lack of oxygen or classical energy sources [25].

Similarities between TNBC and metastatic cancer cells emerged, relating to metabolites that indicated a shift towards the Warburg effect, as well as the overutilization of nucleotides in PM-M1 for Group A. This overutilization of nucleotides as energy sources increased more in the metastatic cluster than in the TNBC one. This suggests that additional energy pathways from nucleotide catabolism were engaged. In Groups B and C, none of the control cells with higher energy production exhibited elevated nucleotide utilization to generate NADH. In Group B, metastatic lines showed reduced utilization of non-primary energy sources as compared with the TNBC cluster. These observations are consistent with the metastatic activity of these cells, enabling them to travel and successfully colonize distant tissues, suggesting an effective proliferation behavior. TNBC cells tend to be more amorphous, with differential expression and greater diversity in substrate utilization for energy production that is less directed and targeted. Both would have to pivot to anaerobic respiration and rely on it heavily for the high rates of migration and proliferation characteristic of their subtypes. This was shown in Group A on PM-M1 in pyruvic acid for metastatic cells, dextrin for metastatic cells, TNBC, and the overall BC group, but also for lactic acid and malic acid in TNBC and metastatic cells as compared with the control. To support rapid proliferation and prioritization of quantity, this highlighted lactic acid utilization in the data shows anaerobic glycolysis in cancer cells, specifically TNBC and metastatic clusters.

L-malic acid is a ubiquitous organic compound that exists in the Krebs cycle as an intrinsic intermediate, naturally present in all living organisms, that has two carboxylic acid groups attached to it. Normal input of glucose as an energy source yields L-malic acid, and its use from the intermediary form on PM-M1 is also consistent with the natural biological processes of aerobic respiration [26]. D-malic acid is the enantiomer of L-malic acid, which is not biologically active or utilized by humans and is not naturally found in nature. Malate dehydrogenase (MDH) is an enzyme responsible for converting malate to oxaloacetate in aerobic respiration and has been found to contribute to the metabolic plasticity of cancer cells [27]. Comparing the kinetics of MDH isolated from cancerous versus healthy human breast tissue demonstrates that, although the Michaelis–Menten constant K_m_ remains unchanged, the V_max_ of cancer-derived MDH was elevated, indicating a higher tendency for NAD+ and malate generation in cancer cells to support glycolysis and further proliferate [28]. The TNBC and metastatic clusters produced significantly more NADH than controls in the presence of both malic acid forms, providing evidence of a metabolic rewiring in which cancer cells adapt to utilize alternative carbon sources to continue growing and proliferating.

**Figure 3:**
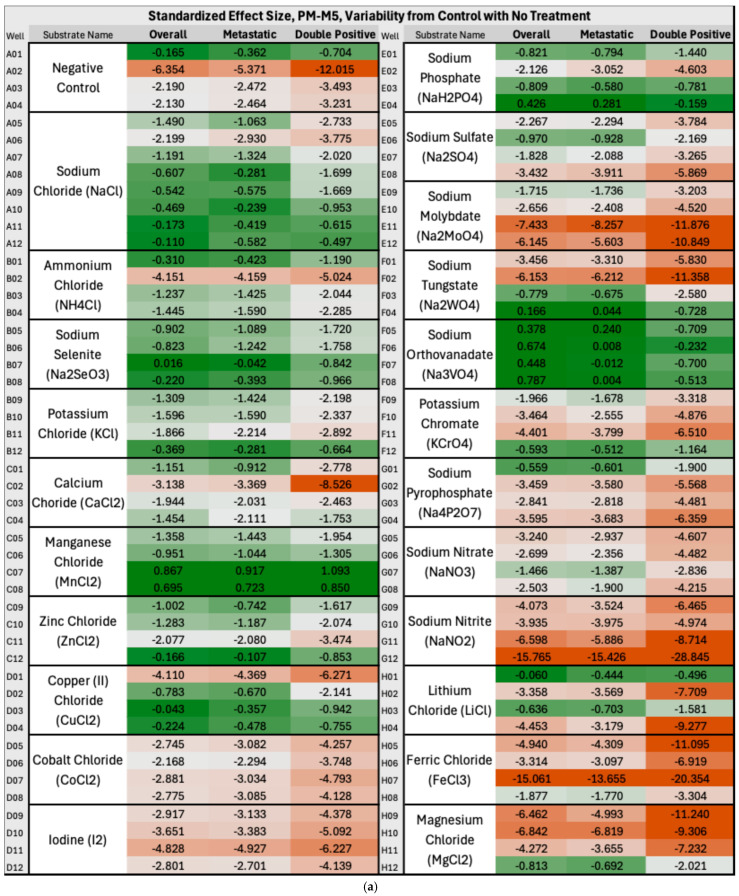

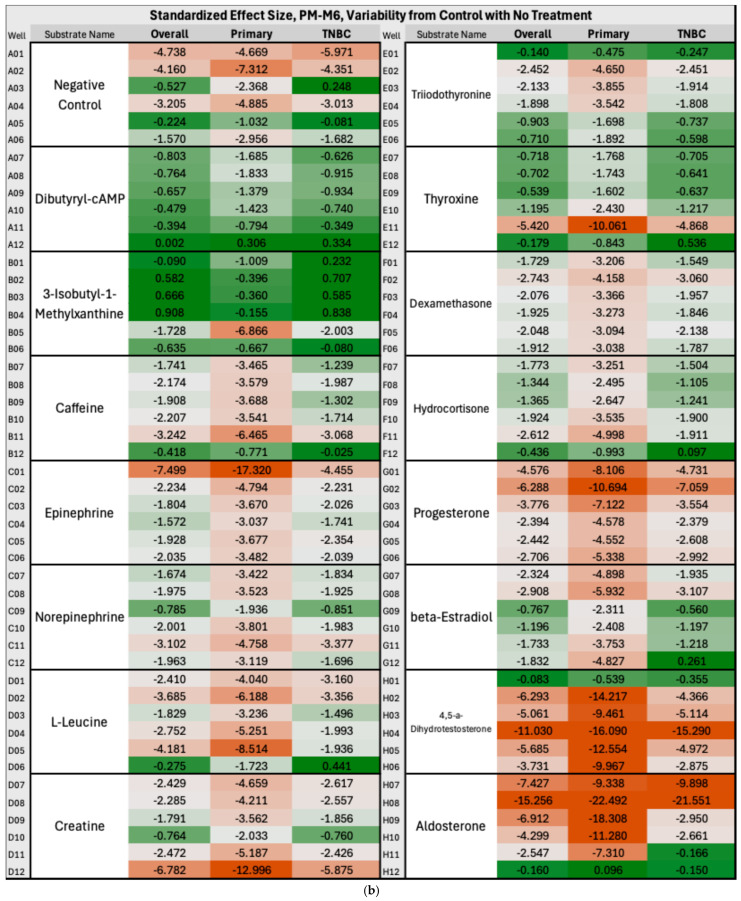
(**a**) **Heatmap of PM-M5 (Group A) depicting standardized effect sizes for NADH energy production in overall, metastatic and double-positive cancer cell subgroups with no treatment in an ionic environment**. The values reflect per-well deviations normalized to control variability, with red indicating decreased energy production and green indicating increased energy production (in the cancer cells as compared with control cells). To calculate the fold-change values, the difference between the mean absorbance generated by NADH levels in cancer and control cells (derived from triplicate measurements) was calculated and then divided by the standard deviation of the control absorbance (derived from triplicate measurements of two control cell lines). Heatmap coloration was applied using a percentile-based three-color scale for all wells and clusters, with the 5th percentile minimum set as dark red, the 50th percentile as light grey, and the 95th percentile maximum as dark green. (**b**) **Heatmap of PM-M6 (Group A) depicting standardized effect sizes for NADH energy production in overall, primary and triple-negative (TNBC) cancer cell subgroups with no treatment in a hormonal and metabolic signaling environment**.

In Group A, the overall BC produced significantly lower NADH levels than controls in approximately one-third of PM-M5 wells, indicating a largely impaired ability of the breast cancer cells to produce energy and thrive in certain ionic environments. This represents a potential therapeutic target for breast cancer overall, and more specifically for ER+/PR+ subtypes, which were heavily disrupted on this plate but not on any other plates containing metabolic effectors (PM-M5 to M8). Ions such as sodium nitrite, iodine, or magnesium chloride appeared particularly effective in halting the metabolism of breast cancer cells and may be considered for further investigation to develop novel treatment approaches. Figure 3a below illustrates the decrease in NADH production by breast cancer cells compared with control cells in the presence of these ions across all the mentioned clusters. In the heatmap coloration, this decrease, corresponding to the aforementioned ions, is represented by boxes that are predominantly red-tinted (wells G9–G12, D9–D12, and H9–H12). Manganese chloride stood out starkly against the trend in plate PM-M5 for overall BC, metastatic, and ER+/PR+ clusters: it was the only ionic species that induced higher NADH production in cancer cells than in controls. Extremely cytotoxic effects were observed in the wells containing high concentrations of this ion; therefore, it would not be considered a likely candidate for therapeutics. As Figure 3 uses a color gradient for the heatmap that only centralizes the middle 95th percentile of the values on PM-M5 and PM-M6 in Group A, the extremes, such as manganese chloride, are not seen below. We primarily focused on the fold change in energy production, aside from outliers. Previous studies have reported similar findings to ours on manganese chloride, specifically corroborating this observation. One paper noted cytotoxicity in human lymphocytes at multiple phases of the cell cycle and DNA damage in G2 following treatment with manganese chloride [29]. Figure 3b depicts a notable increase in energy utilization in primary and TNBC cancer cells relative to control cells under a hormonal and metabolic signaling environment. The most pronounced fold-change increases (shown with darker green shades) in NADH production by cancer cells were observed in wells containing cAMP-elevating modulators, specifically dibutyryl-cAMP (A7–A12) and 3-isobutyl-1-methylxanthine (B2–B6), showing an enhanced metabolic responsiveness in these lines to metabolic effectors.

The metabolic differences with control cells were expected to be milder in primary than in metastatic breast cancer cells, considering how they exhibit greater morphological similarity to non-cancerous cells and tend to maintain most of the functional characteristics of specialized breast tissue. On the other hand, the metastasized cells have colonized a tissue with different features from the primary site and are likely further along in proliferation and “dedifferentiation”; therefore, it is plausible that the metabolic profile may present more marked distinctions from control breast cells and that these cancer cells would be more able to hijack and scavenge energy sources for essential cell metabolism, likely having turned off any cell signaling or extra processes that were not critical for growth and survival.

From the data presented, subtype and stage-specific metabolic fingerprints emerge clearly and can help guide future therapeutic tailoring. While these findings are derived from in vitro cells and should be interpreted with caution, they do suggest that certain subtypes have exploitable metabolic vulnerabilities (i.e., to specific ionic conditions) and could inform future strategies aimed at microenvironmental modulation. The data on cancer cells across the board show the widespread cytotoxic effects of doxorubicin in Groups B and C, targeting cancer and normal cells indiscriminately without a specific metabolic target. Particularly, the analysis in Group C, where both cancer and control cells were treated with doxorubicin, revealed widespread cell death, having little to no metabolic activity in all treated cells. There was indeed little metabolic activity that was subgroup-specific, with significant energy production in the control lines as compared with the primary and ER+/PR+ cancer cells. Even then, the results from the overall cluster for Group C indicated toxic effects on both cancer and healthy cells. It was expected that the doxorubicin-treated breast cancer cell lines would decrease in energy and metabolic production across the board and do the same with high toxicity for the cancer cells as compared with the treated control cells. Doxorubicin decreases energy and metabolic production by inhibiting topoisomerase II. By targeting topoisomerase II, DNA stability is disrupted, which triggers cell cycle arrest [30]. Doxorubicin also triggers redox recycling within the mitochondria, producing superoxide and hydrogen peroxide, which impair mitochondrial enzymes, leading to decreased metabolic production [31]. While baseline profiles of untreated breast cancer cells exhibit subtype-specific metabolic vulnerabilities, exposure to doxorubicin induced largely non-selective metabolic cytotoxicity, underscoring the need for precision strategies that factor in metabolic alterations unique to cancer subtypes. These results reiterate the limitations and adverse effects associated with doxorubicin-based protocols in clinical settings and reinforce the need for effective therapeutic approaches without the detrimental side effects that have been reported with the administration of traditional chemotherapeutics in breast cancer patients.

## 5. Conclusions

Our study delineates distinct metabolic traits in different types of breast cancer cells that may represent novel targets for future drug development. By characterizing the indiscriminate cytotoxicity of the chemotherapeutic agent doxorubicin in both cancer and normal breast cells, we underscore the urgent need for more selective strategies that exploit metabolic distinctions rather than broadly impair cellular viability. These findings provide a foundation for the rational design of targeted metabolic interventions aimed at improving efficacy while minimizing systemic toxicity in breast cancer treatment.

## Figures and Tables

**Table 1:** Characteristics for reference encompassing all human breast lines used in the study.

Human Cell Line	ATCC Accession Number	Morphology	Growth Properties	Tissue	Origin/ Derivation	Tumorigenic	ER Status	PR Status	HER2 Status	Growth Atmosphere
MCF-12A	CRL-3598	Epithelial	Adherent	Breast, mammary gland	Patient with fibrocystic breast disease, prominent regions of intraductal hyperplasia, from adherent population.	No	Normal			5% CO_2_, 95% Air, 37 °C
MCF-12F	CRL-3599	Epithelial	Adherent	Breast, mammary gland	Patient with fibrocystic breast disease, prominent regions of intraductal hyperplasia, from floating population.	No	Normal			5% CO_2_, 95% Air, 37 °C
HCC-38	CRL-2314	Epithelial	Adherent	Primary, breast, duct, mammary gland	Patient with breast cancer, TMN Stage IIB, Grade 3, 3/28 lymph node metastasis. Prior history of leiomyosarcoma.	Yes	(−)	(−)	(−)	5% CO_2_, 95% Air, 37 °C
BT-483 ^1^	HTB-121	Epithelial	Adherent	Primary, breast, mammary gland	Patient with breast cancer, papillary invasive ductal tumor.	Yes	(+)	(+)	(−)	5% CO_2_, 95% Air, 37 °C
MDA-MB-231 ^1^	HTB-26	Basal-mesenchymal epithelial	Adherent	Pleural effusion, breast, mammary gland	Patient with breast cancer. Derived from adenocarcinoma.	Yes	(−)	(−)	(−)	100% Air, 37 °C
MCF-7 ^1^	HTB-22	Luminal A epithelial	Adherent and/or suspension	Pleural effusion, breast, mammary gland	Patient with breast cancer. Derived from adenocarcinoma.	Yes	(+)	(+)	(−)	5% CO_2_, 95% Air, 37 °C

^1^ Note. Information contained in the table for all cell lines was obtained from ATCC [20]. The three cell lines denoted by the footnote also include information regarding subtype classification, morphology, and hormone receptor status, adapted from Subik K. et al. [21].

**Table 2:** Summarization of clusters used for data analysis to generalize metabolic signatures and response to chemotherapeutics for multiple breast cancer subtypes.

Cluster	Rationale for Clustering	Cancer Lines Included
Overall representative breast cancer profile (overall BC)	Covers all available BC lines to make generalized conclusions about metabolic signatures across multiple subtypes with diverse molecular features.	HCC-38, BT-483, HTB-26, MCF-7
Primary breast tumor-derived lines (primary)	These cell lines are derived from primary tumors of breast carcinomas. Representative of the first site of carcinogenesis.	HCC-38, BT-483
Metastatic-origin breast lines (metastatic)	These cell lines are derived from pleural effusions and metastatic sites to understand the metabolic shift at distant locales.	HTB-26, MCF-7
Basal-like/low-differentiated TNBC (TNBC)	Hormone receptor negative, to observe metabolic adaptations in TNBC lines that are poorly differentiated and invasive.	HCC-38, HTB-26
Strong hormone receptor positive lines (ER+/PR+)	Estrogen and progesterone receptor-positive, classically luminal lines that respond to endocrine therapies.	BT-483, MCF-7

## Data Availability

The original contributions presented in this study are included in the article/Supplementary Material. The data are available within the main text and the Supplementary Files. Further inquiries can be directed to the corresponding authors.

## Supplementary Materials

The following supporting information can be downloaded at https://zenodo.org/records/17475784 (accessed on 23 December 2025), Group A (Highlighted analysis. A.) complete analysis of all clusters PM-M1 through PM-M8; Group B (Highlighted analysis. B.) complete analysis of all clusters PM-M1 through PM-M8; Group C (Highlighted analysis. C.) complete analysis of all clusters PM-M1 through PM-M8. The Supplementary Materials accompanying this manuscript contain the complete raw and processed datasets generated from the Biolog PM-M1 through PM-M8 phenotype microarrays across all breast-cancer subtypes and control lines, including untreated and doxorubicin-treated conditions. These files are provided in Excel format to preserve full numerical detail and maintain compatibility with common data-analysis platforms. Each supplementary file is labeled according to the specific group, comparison, and plate type (e.g., “Highlighted analysis. A. Triple Negative PMM1-8_No Treatment_HCC38_HTB26_VS_All Control.xlsx”) and each sheet within details the plate number. The grouping organization (A, B, and C) is described in the text and depicted in Figure 1. Each file includes raw absorbance values, normalized mean values, unadjusted *p*-values, Benjamini–Hochberg adjusted *p*-values, and entries highlighted in green, indicating metabolites that meet the FDR significance threshold. This manner of formatting enables readers to sort, filter, and reanalyze the data directly, without losing any information, ensuring full reproducibility. All files correspond directly to comparisons described in the Section 3.
